# Successful Pregnancy and Delivery After Detecting an Exceptionally Early-Formed Implantation Window Using Endometrial Receptivity Analysis: A Case Report

**DOI:** 10.7759/cureus.85368

**Published:** 2025-06-04

**Authors:** Hiromi Fujiki, Nanako Sato, Atsuko Sugita, Tomoko Murata, Yasutaka Murata

**Affiliations:** 1 Reproductive Medicine, Fertility Center, ART Clinic Mirai, Okazaki, JPN

**Keywords:** endometrial receptivity analysis, implantation window, progesterone exposure, successful pregnancy and delivery, window of implantation

## Abstract

This is a report of an extremely early implantation window identified by endometrial receptivity analysis (ERA A1), in which a blastocyst was transferred onto the endometrium, resulting in a successful pregnancy and birth. An infertile couple, a 35-year-old man and a woman with nulligravida, nullipara, presented to our outpatient department with a history of 10 failed timed intercourse cycles and four unsuccessful intrauterine inseminations (IUIs) over two years. The patient exhibited ovulatory dysfunction, irregular menstruation, and polycystic ovarian syndrome. Despite initial ovarian stimulation and retrieval of good-quality blastocysts, repeated embryo transfer attempts were either canceled due to inadequate endometrial thickness or resulted in implantation failure. Consequently, an ERA was performed. Persistent ERA tests, conducted five times following repeated post-receptive results, ultimately detected an exceptionally early implantation window occurring just 67 hours after progesterone exposure. ERA-guided embryo transfers enabled implantation, but repeated attempts resulted in miscarriage, chemical pregnancy, or failure to implant. After additional oocyte retrieval and 11 embryo transfer attempts, the patient achieved a successful pregnancy and delivered a healthy baby at term. To the best of our knowledge, this case presents the world’s earliest documented window of implantation with a subsequent live birth. It underscores the existence of scenarios where the timing of implantation may occur at unexpected intervals, significantly deviating from expectations based on prior knowledge and clinical experience. This report highlights the importance and practical value of ERA in managing such cases.

## Introduction

A recognized cause of repeated implantation failure (RIF) is a shifted window of implantation (WOI) in in vitro fertilization (IVF) [1]. The human uterus has a specific WOI typically lasting about 24 to 48 hours, during which it can accept an embryo. However, WOI’s exact timing and duration may vary depending on individual and hormonal environments [2]. The endometrial receptivity analysis (ERA) test was recently developed to determine whether the endometrium (EM) is in a receptive or non-receptive state by examining the mRNA expression patterns of 248 genes [3]. While the debate about its optimal use and overall utility is ongoing, the test can be a powerful tool in cases where a displaced WOI is suspected to underlie RIF [4-6]. Here, we report a case of an exceptionally early WOI that ultimately led to a successful pregnancy and delivery. This case has significant implications for future IVF strategies, particularly in managing implantation failures and individualizing the timing of embryo transfer.

This article was previously posted on Research Square on September 9, 2024.

## Case presentation

A 35-year-old patient with nulligravida, nullipara, and height of 158 cm, weight of 54.2 kg, BMI of 21.7, and anti-Mullerian hormone (AMH) level of 14.31 ng/mL, was referred to our clinic after the failure of 10 timed intercourse cycles and 4 intrauterine insemination (IUI) attempts over two years. The patient exhibited irregular menstruation, ovulatory dysfunction, FSH<LH, and polycystic ovarian syndrome [7]. One timed intercourse cycle and two IUI cycles were conducted at our clinic without success, leading to the decision to pursue assisted reproductive technology (ART). Ovarian stimulation with daily FSH injections (total FSH =1575 units) resulted in the retrieval of 30 eggs, 25 metaphase II stage (MII) oocytes, and 20 fertilized embryos (2PN), with 15 embryos reaching the blastocyst stage; 14 good-quality blastocysts were frozen.

First embryo transfer plan: canceled

During the hormone replacement therapy (HRT) cycle, after 15 days of transdermal estradiol (ESTRANA®Tape0.72mg; Hisamitsu Pharmaceutical, Japan) application with an E2 level of 584.7 pg/mL and endometrial thickness of 5.0 mm, additional estradiol tablets (Julina tablets 0.5mg; Bayer Pharmaceutical, Japan) were administered. By cycle day 22, the endometrial thickness was only 5.2 mm, and therefore, the embryo transfer was canceled.

Embryo transfer attempt no. 1

After 15 days of transdermal estradiol application with an E2 level of 842.0 pg/mL and endometrial thickness of 5.3 mm, additional estradiol tablets were administered in the HRT cycle. By cycle day 20, the E2 level was 880.6 pg/mL and the endometrial thickness was 6.4 mm. After 102 hours of progesterone exposure (Lutoral®, Chrolmadinone acetate, 6 mg orally and Onecrinone®, progesterone gel, 90 mg vaginally) on cycle day 25, a 4AA day 4 blastocyst was transplanted. Serum hCG level was measured on cycle day 34 at 0.1 mIU/mL, indicating an unsuccessful transfer.

Embryo transfer attempt no. 2

Following the same protocol as the first embryo transfer attempt, on cycle day 14, the E2 level was 422.9 pg/mL and the endometrial thickness was 7.8 mm; progesterone administration was started on the next day. On cycle day 19, a 4AA day-4 blastocyst was transferred following 102 hours of progesterone exposure. However, on cycle day 28, serum hCG concentration was 0.1 mIU/mL, indicating no pregnancy.

Embryo transfer plan no. 3: canceled

In the HRT cycle with transdermal estradiol, the E2 level was 351.6 pg/mL, and the endometrial thickness was 6.5 mm, so the transfer was postponed. By cycle day 22, the endometrial thickness was only 5.0 mm, leading to cancellation of the embryo transfer.

Intervention: ERA test

After two failed embryo transfers with two good-quality embryos, an ERA test was recommended. During the ERA mock cycle, transdermal estradiol was initiated and gradually adjusted starting at the perimenstrual period. Approximately two weeks later, after confirming the serum estradiol level at 395.6 pg/mL and progesterone level below 1 ng/mL and ensuring that the endometrial thickness reached a minimum of 7 mm, vaginal progesterone and oral progestin were initiated. The ERA biopsy was performed at 127 hours of exogenous progesterone exposure, and the report suggested a post-receptive result.

Following the recommendation in the ERA report, the second biopsy was performed 102 hours after starting exogenous progesterone, following the same protocol as the first biopsy. The ERA report showed a non-informative result, and the time of embryo transfer was not recommended. Therefore, a third biopsy was performed 103 hours after starting exogenous progesterone administration, resulting in another post-receptive result. Following ERA recommendations, a fourth biopsy was performed 24 hours earlier than the third biopsy.

The fourth biopsy, obtained after 78 hours of progesterone exposure as advised in the latest report, suggested a post-receptive result, resulting in concerns regarding the patient's heightened sensitivity to the progesterone formulation utilized thus far. Consequently, both the progesterone brand and route of administration were modified in the fifth ERA mock cycle, transitioning exclusively from a combination of vaginal and oral administration to vaginal administration (Lutinus®, Ferring Pharm, 300 mg per day).

**Figure 1:**
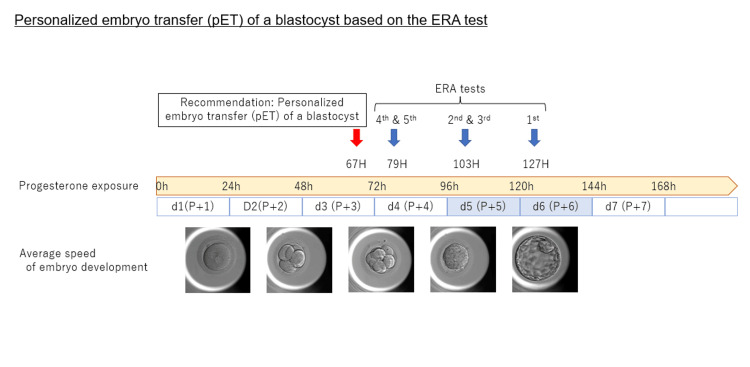
Timeline schema of repeated ERA tests and progesterone exposure intervals This figure illustrates the timing of progesterone exposure and the scheduling of ERA tests and personalized ET. This is the original image created by the author. ERA: endometrial receptivity analysis; pET: personalized embryo transfer

**Figure 2:**
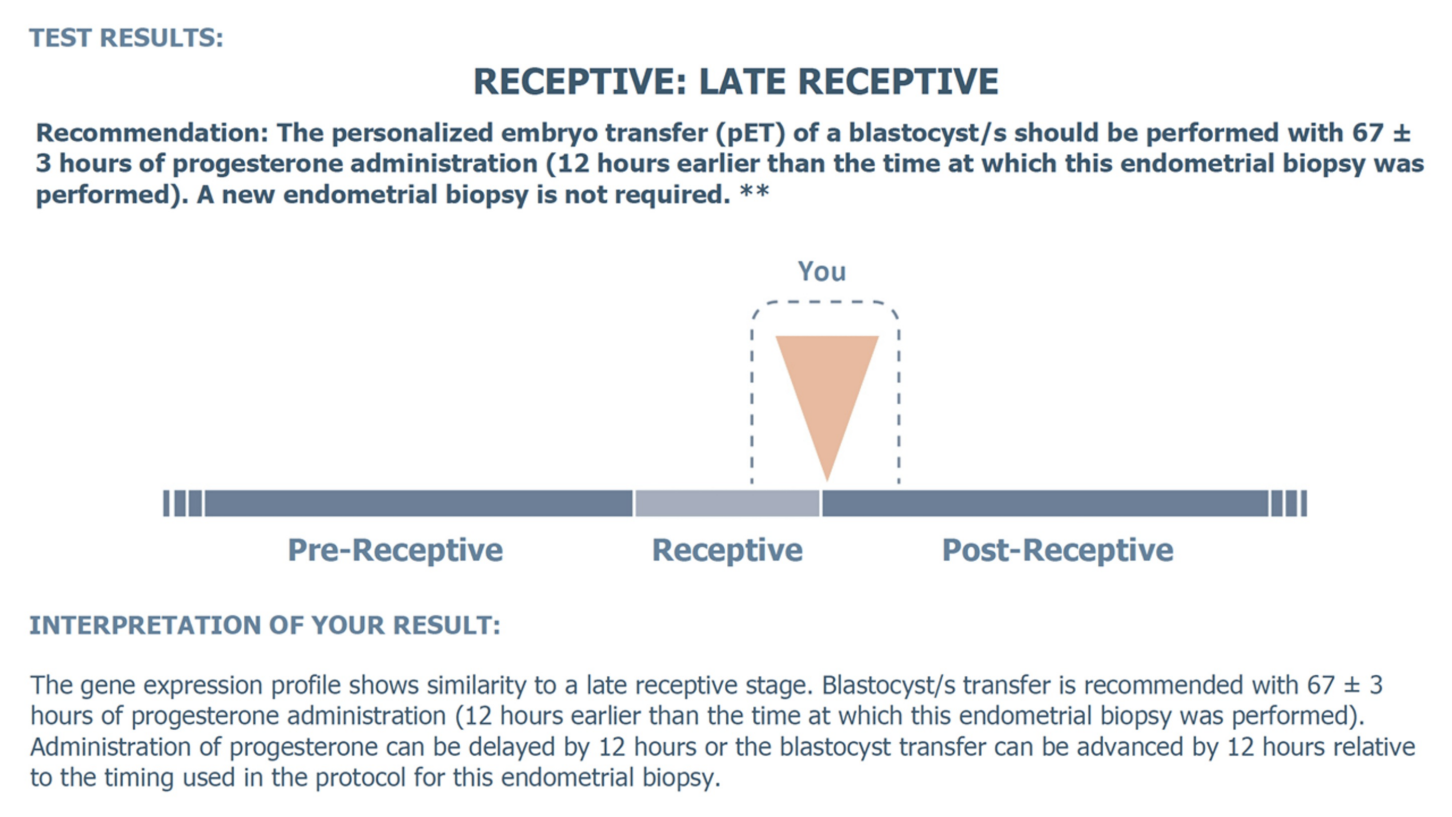
Fifth ERA test result report by Igenomix This figure, created by Igenomix, displays the comprehensive results from the fifth ERA test, detailing findings and clinical recommendations for embryo transfer timing based on the test results. ERA: endometrial receptivity analysis

The fifth biopsy, conducted at 79 hours of progesterone administration, suggested a late receptive result. Ultimately, the final report recommended that the embryo be transferred at 67±3 hours of progesterone exposure (Table 1, Figures 1, 2).

**Table 1:** Detailed overview of sequential ERA tests This table provides a detailed overview of each ERA test conducted, including biopsy timing, type of progesterone used, test results, and subsequent recommendations for further testing or adjustments. ERA: endometrial receptivity analysis; EM: endometrium; E2: estradiol

ERA test	Biopsy timing	peak E2 (pg/mL)	EM thickness (mm)	Type of progesterone	Test result	Recommendation
1st	127 hrs	395.6	5.8	Lutoral®/Onecrinone®	Post-receptive	New endometrial biopsy 1 day earlier
2nd	103 hrs	355.7	5.3	Lutoral®/Onecrinone®	Non-informative	New endometrial biopsy at the same time
3rd	103 hrs	580.8	5.7	Lutoral®/Onecrinone®	Post-receptive	New endometrial biopsy 1 day earlier
4th	78 hrs	339.2	5.7	Lutoral®/Onecrinone®	Post-receptive	Mothe dify progesterone brand and route new endometrial biopsy at the same timing
5th	79 hrs	424.0	6.8	Lutinus®	Late receptive	Transfer at 67 hrs after Progesterone exposure

Embryo transfer attempt no. 3

In the HRT cycle using a gradually increasing transdermal estradiol formulation, a day-5 blastocyst (4AA) was transplanted 67 hours after starting vaginal progesterone administration. An hCG level of 125.7 mIU/mL was detected nine days post-transfer, confirming the presence of a gestational sac on the fifth week of pregnancy. Unfortunately, this led to a spontaneous miscarriage on the fifth week and third day of pregnancy.

Embryo transfer attempt no. 4

Embryo transfer at 67 hours of progesterone exposure was attempted again. Following the same protocol as in embryo transfer attempt No. 3, a blastocyst (4AA) was transplanted 67 hours after initiating vaginal progesterone. Subsequent hCG level measurements revealed 71.4 mIU/mL, 816.7 mIU/mL, and 5221.0 mIU/mL concentrations at three weeks and six days, four weeks and six days, and five weeks and five days of pregnancy, respectively, confirming the presence of a gestational sac. However, despite this confirmation, no heartbeat was detected at eight weeks and one day, leading to a spontaneous miscarriage in week nine.

Embryo transfer attempt no. 5

In another HRT cycle, using a gradually increasing transdermal estradiol formulation, on the 17th day following initiation of the cycle, with an E2 level of 589.7 pg/mL and an endometrial thickness of 4.3 mm, estradiol tablets were introduced. By the 23rd day, with the endometrial thickness reaching more than 6 mm, a day-5 blastocyst (4AA) was transplanted 67 hours after the first vaginal progesterone dose. The hCG level was 0.1 mIU/mL at nine days post-transfer, indicating another unsuccessful transfer.

Embryo transfer attempt no. 6

In a repeat HRT cycle, using a gradually increasing transdermal estradiol formulation, by the 18th day of the cycle, with an E2 level of 678.6 pg/mL and an endometrial thickness of 6.0 mm, two day-5 blastocysts (4AA and 4BA) were transferred 67 hours after initiating vaginal progesterone. The hCG level was 0.1 mIU/mL at nine days post-transfer.

Embryo transfer attempt no. 7

The same HRT cycle protocol as in embryo transfer attempt no. 6 was followed. On the 16th day of the cycle, with an E2 level of 449.9 pg/mL and an endometrial thickness of 6.7 mm, two day-5 blastocysts (4AA) were transplanted 66 hours after the initiation of vaginal progesterone. The hCG level was 10.2 mIU/mL at four weeks, indicating a chemical abortion.

Embryo transfer plan no. 8: canceled

Starting a new HRT cycle with a gradually increasing transdermal estradiol formulation, estradiol tablets were introduced on the 16th day of the cycle, coinciding with an E2 level of 840.7 pg/mL and an endometrial thickness of 5.9 mm. However, by the 20th cycle day, despite the E2 level maintained at 750.6 pg/mL, the endometrial thickness decreased to 5.2 mm, and so the embryo transfer was cancelled.

Embryo transfer attempt no. 8

In this HRT cycle using a gradually increasing transdermal estradiol formulation, with an endometrial thickness of 5.7 mm and an E2 level of 1032.0 pg/mL on the 18th day, progesterone was initiated on the next day. Two-day-5 blastocysts (4AA and 4AB) were transferred at 66 hours of exogenous progesterone exposure. The hCG level was 94.56 mIU/mL at four weeks and 1251.0 mIU/mL at five weeks and one day. Although a gestational sac was confirmed, no heartbeat was detected at five weeks and one day, leading to a spontaneous miscarriage at six weeks and five days.

Embryo transfer attempt no. 9

In the HRT cycle using a gradually increasing transdermal estradiol formulation, on the 17th day, with an E2 level of 406.3 pg/mL and an endometrial thickness of 9.0 mm, two day-5 blastocysts (4AA and 4BA) were transferred 66 hours after the first dose of vaginal progesterone. The hCG level was 0.1 mIU/mL at four weeks.

Following repeated thawed embryo transfers with poor progress, the patient decided to undergo an ERA in a natural cycle. The ERA, performed at 88 hours after ovulation, revealed a late receptive window of implantation. The recommended transplantation time was identified at 76±3 hours after hCG administration.

Embryo transfer attempt no. 10

When attempting the embryo transfer in a natural cycle, the E2 level was 605.9 pg/mL, and the endometrial thickness was 7.2 mm on day 14 of the cycle. On cycle day 17, two blastocysts (4AB and 4BC) were transferred 76 hours after hCG injection. The hCG level on cycle day 26 was 0.5 mIU/mL.

After retrieving the additional ova, the patient proceeded with another embryo transfer cycle.

Embryo transfer attempt no. 11

In an HRT cycle using transdermal estradiol, after 20 days of endometrial priming, with an E2 level of 795.8 pg/mL and an endometrial thickness of 6.8 mm, two blastocysts (4AA) were transferred on day 23 of the cycle following 67 hours of exogenous progesterone exposure. The hCG level at four weeks and one day of pregnancy was 189.8 mIU/mL, and a gestational sac was confirmed.

The patient was transferred to a perinatal care facility at 10 weeks and three days of pregnancy and she delivered a baby vaginally at 40 weeks and four days, weighing 3316 g (Table 2).

**Table 2:** Detailed overview of sequential embryo transfer and pregnancy outcomes EM: endometrium; E2: estradiol

＃of ET	peak E2 conc (pg/mL)	EM thickness (mm)	Embryo grade	hCG value at pregnancy test	Pregnancy outcome
1st	880.6	6.4	4AA (day 4)	0.1 (mIU/mL)	Not pregnant
2nd	422.9	7.8	4AA (day 4)	0.1	Not pregnant
ERA	Intervention				
3rd	521.6	7.5	4AA (day 5)	125.7	Spontaneous abortion
4th	828.6	6.2	4AA	71.4	Spontaneous abortion
5th	1237.0	9.7	4AA	0.1	Not pregnant
6th	678.6	6.0	4AA,4BA	0.1	Not pregnant
7th	449.9	6.7	4AA,4AA	10.2	Chemical abortion
8th	1032.0	5.7	4AA,4AB	94.56	Spontaneous abortion
9th	406.3	9.0	4AA,4BA	0.1	Not pregnant
10th	605.9	7.2	4AB,4BC	0.5	Not pregnant
11th	795.8	6.8	4AA,4AA	189.8	Get pregnant and delivery

## Discussion

In this case, repeated ERA tests facilitated the identification of an exceptionally early window for implantation following exposure to luteal hormones. Despite initial non-implantation and miscarriages, persistent efforts with repeated transplants ultimately culminated in a successful pregnancy and the birth of a healthy baby.

WOI fluctuates depending on factors such as hormonal environment, the presence or absence of inflammation, and patient age [8]. RIF can occur due to multiple causes, including a shifted WOI. Most patients with recurrent implantation failure are receptive at the expected time [9]. However, which patients should undergo testing, when during treatment it should be performed, and how beneficial it truly is remain debatable [10].

We generally recommend testing after three to four failed implantation attempts at our clinic. In this case, however, the endometrium was extremely thin, and we were concerned about whether the WOI was truly open and whether essential genes were being expressed. Consequently, we advised early testing after just two failed attempts.

Consequently, determining the correct timing of the WOI required repeated tests, leading to considerable costs and time investment. Throughout this process, the couple was explained the importance of the WOI, emphasizing that transferring an embryo outside this window would not result in implantation or pregnancy. We continued testing, along with ongoing counseling, until the recommended timing was identified. Notably, the WOI was approximately 60 hours (about 2-2.5 days) earlier than usual, far exceeding our initial assumptions. Although we proceeded with some uncertainty, we achieved a successful outcome.

To the best of our knowledge, this case presents the earliest documented WOI, at just 67 hours after initiating exogenous progesterone, with a subsequent live birth. It highlights the need for a more nuanced understanding of endometrial receptivity and suggests that clinical use of ERA should account for atypical timing scenarios to improve treatment outcomes.

## Conclusions

In conclusion, this case demonstrates scenarios where the WOI occurs at unforeseen intervals, diverging significantly from expectations based on prior knowledge and clinical experience. It underscores ERA’s importance and practical utility, especially in complex cases where standard protocols are insufficient. Our report emphasizes the need for personalized approaches in reproductive medicine, enhancing the likelihood of successful pregnancy outcomes.
